# Development of a joint diagnostic model of thyroid papillary carcinoma with artificial neural network and random forest

**DOI:** 10.3389/fgene.2022.957718

**Published:** 2022-10-07

**Authors:** Shoufei Wang, Wenfei Liu, Ziheng Ye, Xiaotian Xia, Minggao Guo

**Affiliations:** Department of Thyroid, Parathyroid, Breast, and Hernia Surgery, Shanghai Sixth People’s Hospital Affiliated to Shanghai Jiao Tong University School of Medicine, Shanghai, China

**Keywords:** papillary thyroid carcinoma, random forest, artificial neural network, early diagnostic, biomarker

## Abstract

**Objective:** Papillary thyroid carcinoma (PTC) accounts for 80% of thyroid malignancy, and the occurrence of PTC is increasing rapidly. The present study was conducted with the purpose of identifying novel and important gene panels and developing an early diagnostic model for PTC by combining artificial neural network (ANN) and random forest (RF).

**Methods and results:** Samples were searched from the Gene Expression Omnibus (GEO) database, and gene expression datasets (GSE27155, GSE60542, and GSE33630) were collected and processed. GSE27155 and GSE60542 were merged into the training set, and GSE33630 was defined as the validation set. Differentially expressed genes (DEGs) in the training set were obtained by “limma” of R software. Then, Gene Ontology (GO) and Kyoto Encyclopedia of Genes and Genomes (KEGG) enrichment analysis as well as immune cell infiltration analysis were conducted based on DEGs. Important genes were identified from the DEGs by random forest. Finally, an artificial neural network was used to develop a diagnostic model. Also, the diagnostic model was validated by the validation set, and the area under the receiver operating characteristic curve (AUC) value was satisfactory.

**Conclusion:** A diagnostic model was established by a joint of random forest and artificial neural network based on a novel gene panel. The AUC showed that the diagnostic model had significantly excellent performance.

## Introduction

Thyroid cancer (TC), accounting for 3.4% of all cancers diagnosed annually, is the most common endocrine malignancy (Seib and Sosa, 2019). TC was divided into three types: differentiated thyroid cancer, representing over 90% of thyroid cancer, consists of papillary thyroid carcinoma (PTC) and follicular thyroid carcinoma (FTC). In addition, anaplastic thyroid cancer (ATC, 1%) and poorly differentiated thyroid cancer (PDTC, 5%) are rare tumors (Prete et al., 2020). With the popularization of physical examination, the incidence of PTC has increased rapidly (Wang and Sosa, 2018). PTC is usually an inert and curable tumor with a 10-year survival rate>90% (Alvarado et al., 2009). However, more than 25% of advanced-stage PTC patients are characterized by their invasiveness and metastasis; these traits usually result in poor prognosis (Huang et al., 2021). Among PTC patients, cervical lymph node metastasis (LNM) occurs in 50%–80% of patients, which is a tested risk factor for recurrence and a reduced survival rate (Ling et al., 2021). Distant metastasis occurs in 2% of advanced-stage PTC, which is the main reason for death. Lung is the most common metastatic site (53.4%), and 26.3% presented with the multiple-organ metastasis (Toraih et al., 2021). So, we intended to discover novel and important gene panels and develop a diagnostic model for PTC of early stage.

The availability of microarray technology and more precise RNA-sequencing technology improves the research of disease pathogenesis (Xie et al., 2020). Discovering the most meaningful variables for classification is the primary question about developing a classification model using gene expression data. To resolve this, a variety of machine learning algorithm, including random forest (RF) (Kursa, 2014; Cai et al., 2015) and artificial neural network (ANN) (Chen et al., 2014), were used. Differing from common statistical methods, machine learning involves learning from cases (Van Calster et al., 2019). Therefore, RF and ANN were joined to develop a novel diagnostic model of PTC by learning from the training set and then testing the model in the validation set. The results of this study reveal a novel gene panel for early clinical diagnosis of PTC.

## Materials and methods

### Research design

**FIGURE 1:**
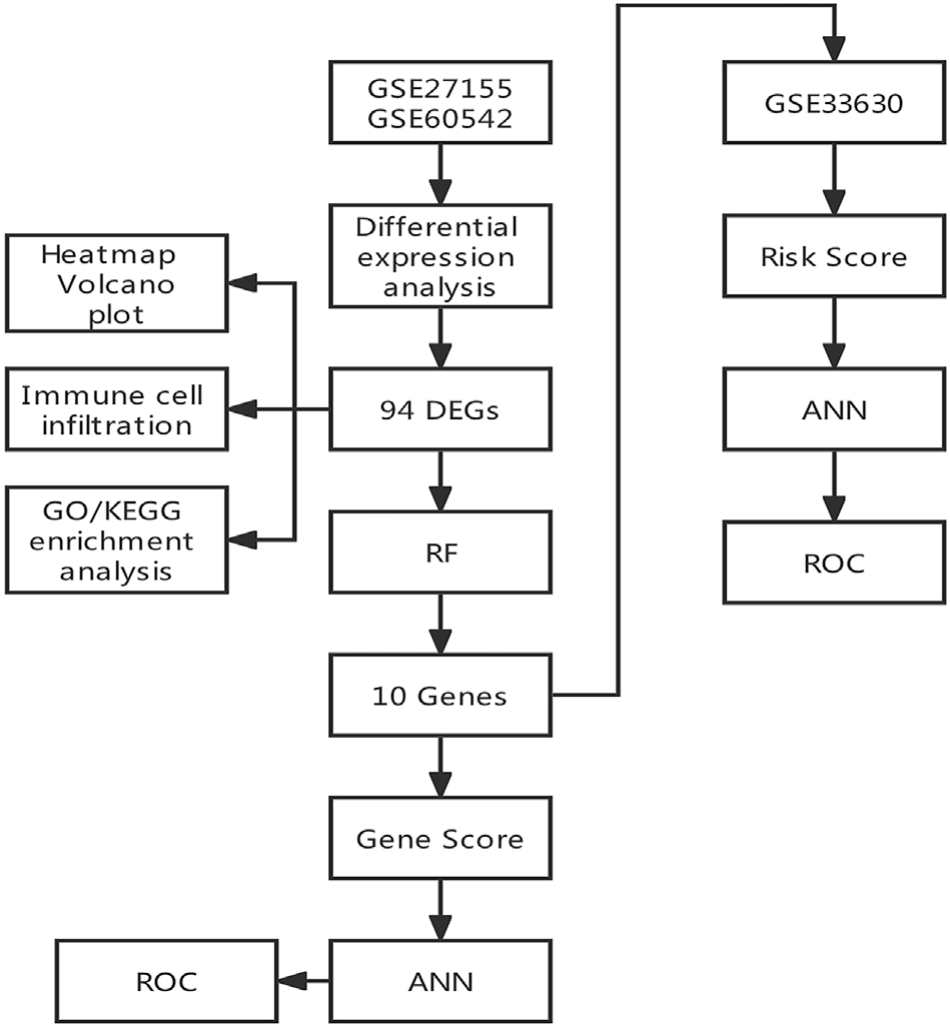
Flowchart.

The study flowchart is shown in Figure 1. Three gene expression datasets (GSE27155, GSE60542 and GSE33630) were collected from the GEO database. GSE27155 and GSE60542 were integrated into the training set, and GSE33630 was selected as the validation set. Differentially expressed genes (DEGs) were defined by the R package “limma.” Gene Ontology (GO) function enrichment analyses and Kyoto Encyclopedia of Genes and Genomes (KEGG) pathway enrichment analyses were conducted through the “clusterProfiler” package of R based on the DEGs in the training set. Also, immune cell infiltration analysis was performed by R. We determined 10 important genes through random forest from the DEGs by the “randomForest” package. Further, an artificial neural network diagnostic model was established through the “neuralnet” package using the 10 important genes and was assessed by AUC. Finally, the validity of the ANN diagnostic model was validated with the performance of a validation set.

### Data download and process

The GSE27155, GSE60542, and GSE33630 datasets were collected *via* the Gene Expression Omnibus database (GEO; https://www.ncbi.nlm.nih.gov/geo/). Then, gene names were obtained by transforming probe names by R software. If several probes could be matched to identical gene, the expression data with gene was replaced by its mean expression value. Finally, 55 samples (4 normal samples and 51 PTC samples) in GSE27155, 63 samples (30 normal samples and 33 PTC samples) in GSE60542 and 94 samples (45 normal samples and 49 PTC samples) in GSE33630 were utilized in this research (Table 1). GSE27155 and GSE60542 were combined into a training set, and GSE33630 as a validation set.

**TABLE 1:** Source of datasets.

Datasets	Platform	Normal	PTC
GSE27155	GPL96	4	51
GSE60542	GPL570	30	33
GSE33630	GPL570	45	49

### Screening for differentially expressed genes

Differentially expressed genes (DEGs) were identified in 34 normal samples and 84 PTC samples of the training set through the R package “limma” using the classic Bayesian data analysis. |log2FC| >1.5 and adjusted *p* Value <0.05 were set as threshold. Then, we obtained 94 DEGs involving 53 up-regulated genes and 41 down-regulated genes. R package “pheatmap” and “ggplot2” were utilized to conduct the heatmap and volcano plot.

### Gene Ontology function and Kyoto Encyclopedia of Genes and Genomes pathway enrichment analysis

To explore the biological significance of these DEGs, Gene Ontology (GO) enrichment analysis (adjusted *p* value < 0.05) categorizing genes into biological process (BP), cellular component (CC), and molecular function (MF) was performed. Meantime, Kyoto Encyclopedia of Genes and Genomes (KEGG) enrichment analysis was used to describe metabolic pathways (*p* value < 0.05). Enrichment analysis of DEGs was conducted through the R package “clusterProfiler.”

### Immune cell infiltration analysis

As a crucial component of the tumor environment, immune cells impacted the development and prognosis of tumors, their composition as well as function in various types of tumors were different. On the one hand, some immune cells have the roles to be favorable targets for immunotherapy. On the other hand, some may react negatively, even resulting in drug resistance. Given these causes, figuring out the ingredient and possible influence of immune cells in PTC was beneficial to identify valuable therapeutic targets. We downloaded a reference including human gene expression in immune cells. Then, we exploited the reference and a file including the gene expression of each sample to complete immune cell infiltration analysis and obtain the result involving the expression of immune cells of each sample by R software. Based on the result, we performed correlation analysis of immune cell by the “corrplot” package and difference analysis of immune cells by the “vioplot” package.

### Random forest analysis to determine important genes

The important genes were identified by a random forest classifier *via* “randomForest” package of R. Firstly, the parameter “ntree” was set as 500 to find the best number of trees. By calculating the error of cross validation, 15 was selected as the best number of trees represented the minimum error of cross validation. Then, parameter 15 was used to reconduct random forest and the importance score of genes was obtained. Genes with an importance score greater than 2 were seen as PTC significantly related genes. The R package “heatmap” was utilized to draw a heatmap based on important genes.

### Development and validation of an artificial neural network model

Based on 10 important genes identified by RF, an artificial neural network model was developed *via* the R package “neuralnet.” At first, the expression of 10 important genes was converted into “gene tag” based on their expression levels. In the case of a certain sample, the expression level of a specific gene was compared to the median of all sample expression level. Among the up-regulated genes, if the expression level is higher than the median, it will be valued as 1, otherwise 0. Among the down-regulated gene, if expression level is higher than median, it will be valued as 0, otherwise 1. Then, we finished a “gene tag” sheet. Next, the hidden layers of ANN were set as 5 to obtain a gene weight calculated by “gene tag”. Finally, the ANN diagnostic model was established. We assessed the model in the training set. Also, we validated the model in the validation set, and its diagnostic performance was evaluated by AUC.

## Results

### Differential expression analysis

**FIGURE 2:**
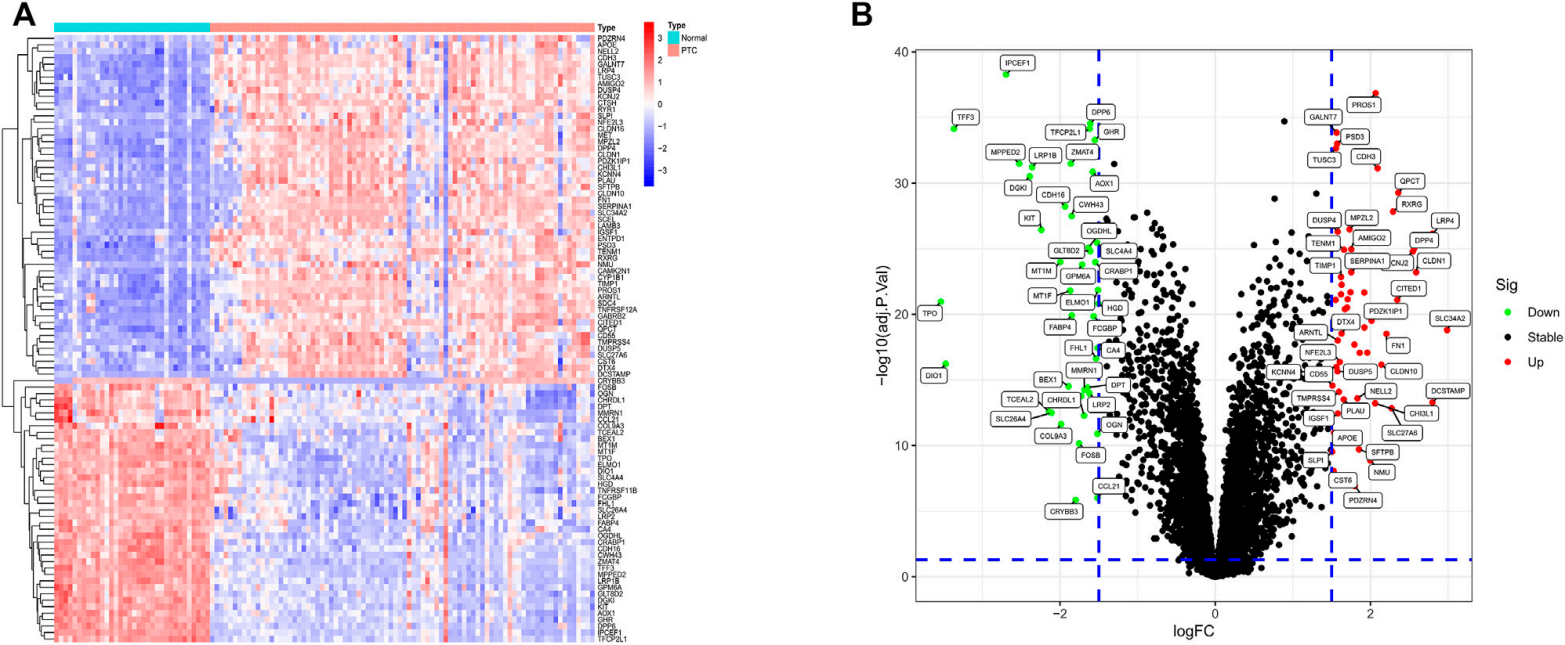
Heatmap and volcano plot of DEGs. **(A)** The heatmap of differential expression analysis result. The colors of the heatmap, from red to blue, indicate high to low expression of genes in normal and PTC samples. On the upper part of the heatmap, the blue band represents normal samples and the red band stands for PTC samples. **(B)** The volcano plot of DEGs. The *X*-axis is logFC, and the *Y*-axis is –log10 (adj.*p* value). The high-expression genes’ adj. *p* value <0.05 and logFC >1.5 are located on the top-right. The down-regulated genes’ adj. *p* value <0.05 and logFC < -1.5 are located on the top-left. In addition, the black dots indicate the remaining stable genes.

In total, 53 significantly up-regulated genes and 41 significantly down-regulated genes were determined between normal samples and PTC samples based on |log2FC| >1.5 and adjusted *p* value <0.05 as threshold in training sets GSE27155 and GSE60542. Heatmaps (Figure 2A) and volcano plots (Figure 2B) of DEGs showed favorable discrimination of gene expression.

### Gene Ontology/Kyoto Encyclopedia of Genes and Genomes enrichment analysis

**FIGURE 3:**
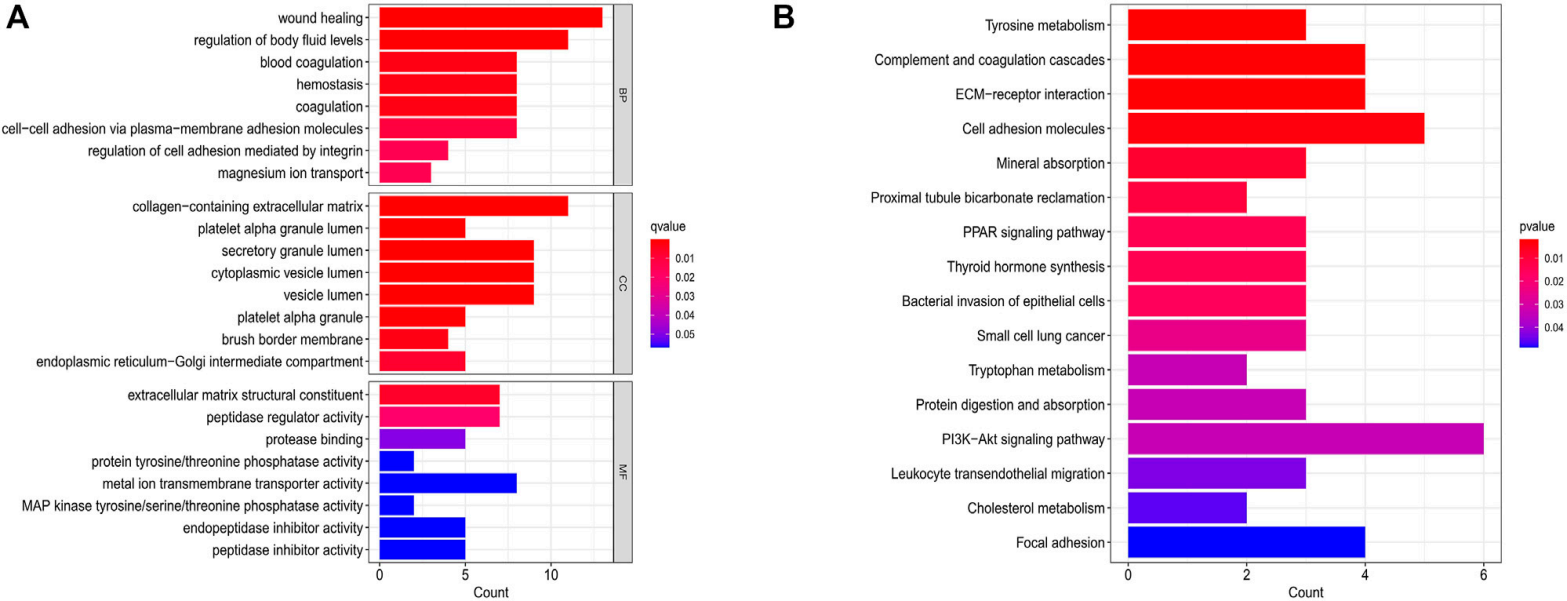
The results of GO and KEGG enrichment analysis. **(A)** The GO function enrichment analysis of DEGs. The *X*-axis stands for count of genes. The *Y*-axis represents BP, CC, and MF. **(B)** The KEGG pathways enrichment analysis of DEGs. The abscissa shows the count of genes, and the ordinate exhibits pathways.

To reveal the biological importance of DEGs in the mechanism of PTC, we conducted GO and KEGG enrichment analysis using the package “clusterProfiler” of R software. Amid GO enrichment analysis results (adjusted *p* value cutoff = 0.05), concerning BP included wound healing, regulation of body fluid levels, blood coagulation and hemostasis; CC involved collagen-containing extracellular matrix; MF contained extracellular matrix structural constituents and other important functions (Figure 3A). KEGG pathway enrichment analysis (*p* value cutoff = 0.05) indicated that the DEGs were significantly related to tyrosine metabolism, complement and coagulation cascades, ECM–receptor interaction, and cell adhesion molecules (Figure 3B).

### Immune cell infiltration analysis

**FIGURE 4:**
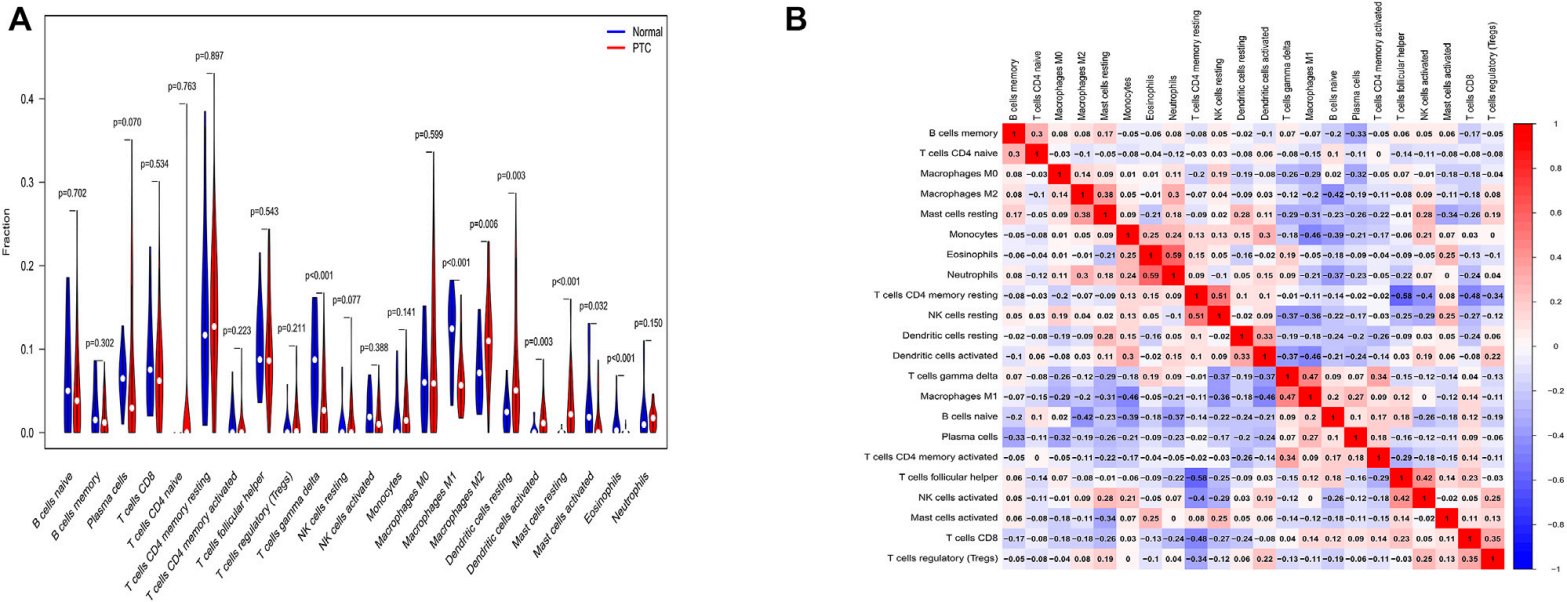
The results of immune cell infiltration analysis. **(A)** The distribution of immune cells between normal samples and PTC samples. The abscissa shows various immune cells, and the ordinate shows fraction. *p* < 0.05 was regarded as statistically significant. **(B)** Correlation analysis of immune cells. The abscissa and ordinate are on behalf of immune cells, and the number stands for correlation. Red color represents a positive correlation, and blue color represents a negative correlation. The larger absolute value or deeper color explains the higher correlation.

We utilized a reference including human gene expression in immune cells and a file involving gene expression of each sample to perform immune cell infiltration analysis. Then, we conducted difference analysis of immune cells by the “vioplot” package and correlation analysis of immune cells by the “corrplot” package. Compared with normal samples, T cells gamma delta, macrophages M2, dendritic cells resting, dendritic cells activated and mast cells resting were higher in PTC samples (*p* < 0.05). Conversely, macrophages M1, mast cells activated, and eosinophils were lower in PTC(*p* < 0.05) (Figure 4A). And macrophages M1 were correlated with dendritic cells activated and T cells gamma delta (correlation, 0.46 and 0.47) (Figure 4B).

### Diagnosis-related important genes with random forest

**FIGURE 5:**
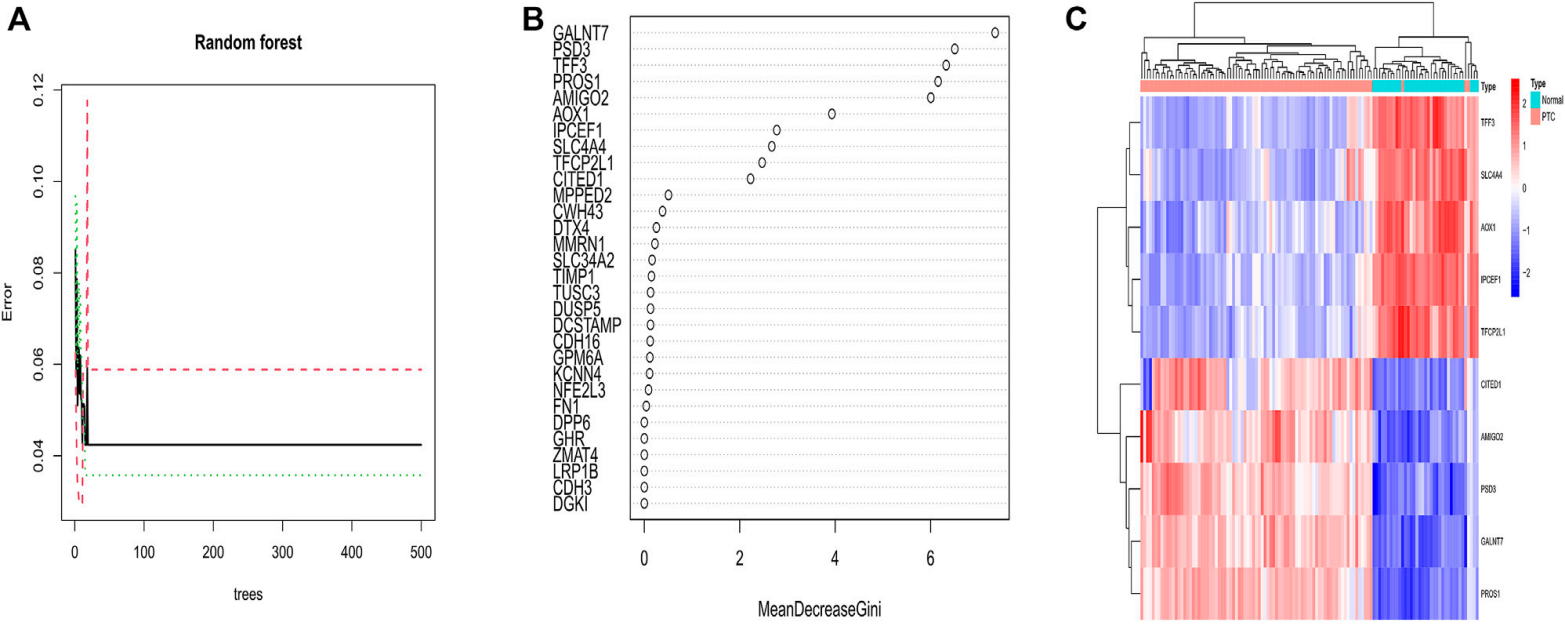
**(A)** Correlation between the number of decision trees and the error of cross validation. The number of decision trees is shown on the abscissa; the error of cross validation is exhibited on the ordinate. The best number of decision trees was 15 for which the error of cross validation was minimum. **(B)** The *X*-axis stands for the importance score of genes calculated by the Gini coefficient method. The *Y*-axis represents the names of genes. **(C)** The heatmap of 10 important genes determined by random forest. Red color represents up-regulated genes in both samples, and blue color is on behalf of down-regulated genes in both samples. Above the picture, PTC samples are showed as a red band, and blue band indicates normal samples.

We performed a random forest based on 94 DEGs to determine important genes. Considering the relationship plot between error of cross validation and the number of decision trees, we chose 15 trees as the parameter of the final model, which revealed a minimum error of cross validation in the model (Figure 5A). Eventually, 10 important genes were determined on the condition that their importance score was greater than 2 for the following analysis (Figure 5B). Amid the 10 important genes, GALNT7 was the most important. With heatmap, 10 important genes could be differed from normal samples and PTC samples. Among them, CITED1, AMIGO2, PSD3, GALNT7, and PROS1 were down-regulated in normal samples and up-regulated in PTC samples. TFF3, SLC4A4, AOX1, IPCEF1, and TFCP2L1 were up-regulated in normal samples and down-regulated in PTC samples (Figure 5C).

### Development and validation of an artificial neural network model

**FIGURE 6:**
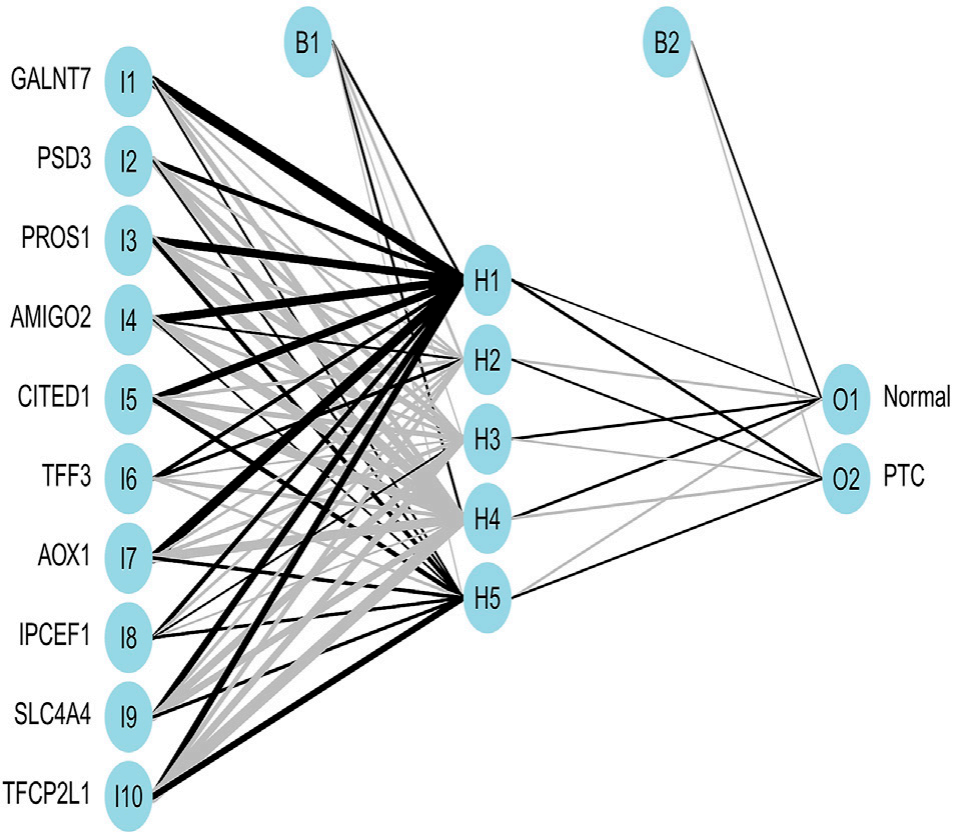
The visualization of the ANN diagnostic model. The neural network topology with 10 input layers consists of important genes; 5 hidden layers; and 2 output layers, including normal and PTC.

**FIGURE 7:**
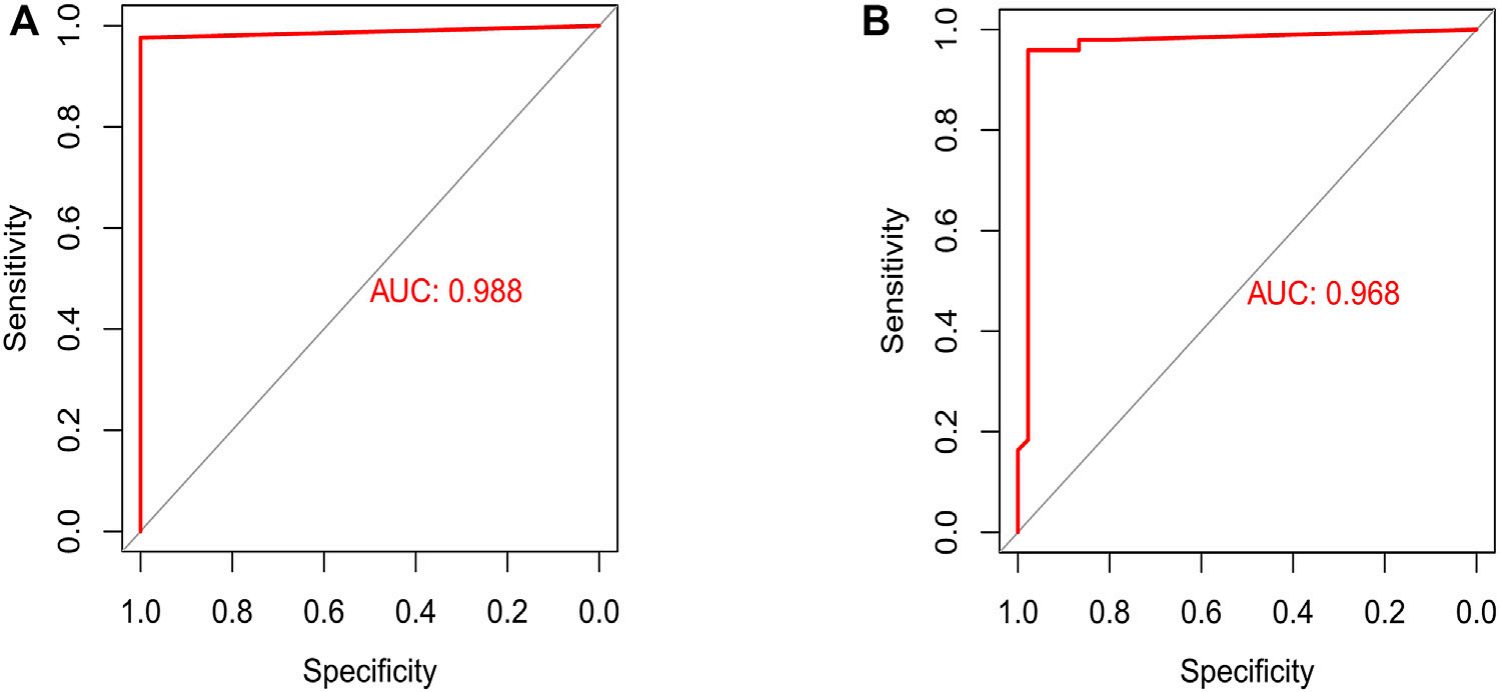
Assessment and testification of the ANN diagnostic model by the ROC curve. **(A)** The assessment result of the training set; **(B)** the testification result of the validation set.

Expression of important genes determined by RF was transformed into “gene tag” marked as 0/1. The weight of all genes was calculated for optimal discrimination between normal samples and PTC samples. Then, an ANN diagnostic model based on gene weight was established (Figure 6). Performance of the model had an AUC of 0.988 (Figure 7A) in the training set and 0.968 (Figure 7B) in the validation set, indicating that the model was very satisfactory in diagnosing PTC. The results demonstrated that we had developed a precise diagnostic model between PTC and normal samples.

## Discussion

To date, employment of machine learning algorithms and wide application of gene expression data in public databases offer methods to find biomarkers for cancer diagnosis in a list of fields (Wang et al., 2018; Tabl et al., 2019). The integration of RF and ANN can be employed to establish a stable diagnostic model for some diseases, like ulcerative colitis and abdominal aortic aneurysm (Li et al., 2020; Duan et al., 2022). Papillary thyroid carcinoma is characterized by slow progression and good prognosis. Early-stage PTC patients have a high postoperative survival rate, but advanced-stage PTC patients still have the risk of lymph node metastasis and distant metastasis, which seriously affect treatment and prognosis. Though ultrasound is viewed as primary screening approach for PTC, the diagnostic accuracy relying on node size is changeable, especially for nodules ≤10 mm (Sutherland et al., 2021). Other factors such as equipment, scan gain, dynamic range, frequency, and doctors also significantly impact the accuracy of ultrasound diagnosis (Gong et al., 2022). Owing to the absence of a perfect diagnostic approach and a lack of potential biomarkers that can be utilized in clinical practice, it is critical to establish a model for early diagnosis and screening of PTC.

Among 10 important genes identified by RF, CITED1, AMIGO2, PSD3, GALNT7, and PROS1 were down-up-regulated in normal samples and up-regulated in PTC samples, TFF3, SLC4A4, AOX1, IPCEF1, and TFCP2L1 were up-regulated in normal samples and down-regulated in PTC samples. Then, an ANN diagnostic model was established, and the performance was assessed by AUC (0.988). Also, we testified the diagnostic ability in the validation set and the AUC was 0.968, which had great efficiency. Together, the developed diagnostic model could offer a novel perspective on our research of the mechanism of PTC.

In a previous study, 9 genes have been verified among 10 important genes related to PTC. The incidence and progression of a series of malignancies are relevant to the high expression of Polypeptide N-Acetylgalactosaminyltransferase7 (GALNT7) and its family members (Nakagawa et al., 2017; Detarya et al., 2020). Wang argued that GALNT7 by activating EGFR/PI3K/AKT kinase pathway to promote cell proliferation and invasion of papillary thyroid cancer (Wang et al., 2021a). In recent years, various human Pleckstrin and sec7 domain-containing 3 (PSD3) have been believed to be related to some tumors, like acute myeloid leukemia (Walker et al., 2021), breast cancer metastasis (Thomassen et al., 2009), astrocytoma progression (van den Boom et al., 2006). PSD3 inhibits apoptosis in papillary thyroid cancer by promoting proliferation, migration, invasion and G1/S transition (Jin et al., 2021). Abnormal expression of Protein S (PROS1) affects human papillary thyroid cancer progression, especially associated with lymph node metastasis (Wang et al., 2021b). Low expression of SLC4A4 affects invasion, metastasis, and the MAPK signaling pathway in PTC. Huang argued the down-regulation of SLC4A4 might be on account of the excessive iodine intakes of patients (Huang et al., 2021). Glu/Asp rich carboxy-terminal domain 1 (CITED1) *via* Wnt/β-catenin signaling pathway results in the development of PTC (Wang et al., 2019). Li showed that CITED1 increased phosphorylation of pRb as well as E2F1 transcriptional activity when p21 and p27 were expressed at low levels, and verified CITED1 was involved in PTC cell proliferation and tumorigenesis (Li et al., 2018). Yu reported that aldehyde oxidase 1 (AOX1) protein level in blood plasma was lower in patients with PTC, which indicated that AOX1 level in blood plasma had the potential to differ in PTC from healthy humans. Furthermore, low levels of AOX1 were highly related to poor survival of PTC (Yu et al., 2021). IPCEF1 was viewed as a significant biomarker for PTC. Moreover, a study showed that the hsa_circ_IPCEF1/hsa-miR-3619–5p axis was associated with the mechanism of PTC, which offers a new idea for further diagnosis and treatment of PTC (Guo et al., 2021). Several researchers revealed noticeable differences in Trefoil factor 3 (TFF3) between benign thyroid nodules and thyroid malignancy (Krause et al., 2008; Karger et al., 2012). Low expression of TFCP2L1 can promote the progression of PTC and CircHACE1 curbs PTC development by upregulating TFCP2L1 through adsorbing miR-346 (Li et al., 2021). Interestingly, we identified another important gene, Adhesion Molecule With Ig Like Domain 2 (AMIGO2), which has never been reported to be associated with PTC.

In addition, the research also has several limitations. Firstly, though we have validated 10 significantly PTC-related genes, the sample size is relatively small. Secondly, the ANN diagnostic model was conducted using datasets from the GEO database, so it should be tested in laboratory experiments and clinical practice.

## Conclusion

In this study, 10 genetic biomarkers related to PTC were determined and the ANN model established by the 10-gene panel displayed satisfactory performance when diagnosing PTC. Moreover, the present research offers a useful basis for early screening of PTC and promotes further study for development of PTC as well as provides potential genes as targets for clinical treatment. In conclusion, our finding has a certain clinical value that can be valuable for early diagnosis of PTC.

## Data Availability

The datasets presented in this study can be found in online repositories. The names of the repository/repositories and accession number(s) can be found in the article/Supplementary Material.
